# A Rare Case of Osteomyelitis of an Ankle Caused by *Mycobacterium chelonae*

**DOI:** 10.3390/antibiotics12010097

**Published:** 2023-01-06

**Authors:** Lenka Ryskova, Rudolf Kukla, Radka Bolehovska, Libor Prokes, Milan Vajda, Tomas Kucera, Ivo Pavlik, Pavel Bostik, Pavel Ryska

**Affiliations:** 1 Institute of Clinical Microbiology, Faculty of Medicine in Hradec Kralove, Charles University and University Hospital, 50005 Hradec Kralove, Czech Republic; 2 Department of Orthopedics, Faculty of Medicine in Hradec Kralove, Charles University and University Hospital, 50005 Hradec Kralove, Czech Republic; 3 Department of Diagnostic Radiology, Faculty of Medicine in Hradec Kralove, Charles University and University Hospital, 50005 Hradec Kralove, Czech Republic; 4 Faculty of Regional Development and International Studies, Mendel University in Brno, tr. Generala Piky 7, 61300 Brno, Czech Republic

**Keywords:** nontuberculous potentially pathogenic mycobacteria, rapidly growing mycobacteria, osteomyelitis

## Abstract

*Mycobacterium chelonae*, a rapidly growing nontuberculous mycobacterium, is usually described as a causative agent of soft tissue infections (postsurgical, posttraumatic, posttransplantation, postinjection, catheter infection, etc.), but only rarely as a cause of osteomyelitis. The authors describe a case report of a 72-year-old man with osteomyelitis of the talus. Initially, the infection was assessed as a soft tissue infection, without any osteolytic changes on the X-ray. After cultivation with subsequent targeted molecular typing of the *rpo*B gene, *M. chelonae* was identified from the affected tissue. The bone involvement was subsequently detected on MRI and confirmed histologically with findings of the granulomatous tissue and acid-fast bacilli. The patient was initially treated intravenously with a combination of tigecycline, amikacin, and moxifloxacin for 4 weeks, after which the oral combination of doxycycline and moxifloxacin continued. Identification of the infecting pathogen using molecular typing thus helped to establish the correct diagnosis and represents a rarely described case of osteomyelitis caused by *M. chelonae.*

## 1. Case Report

**Figure 1:**
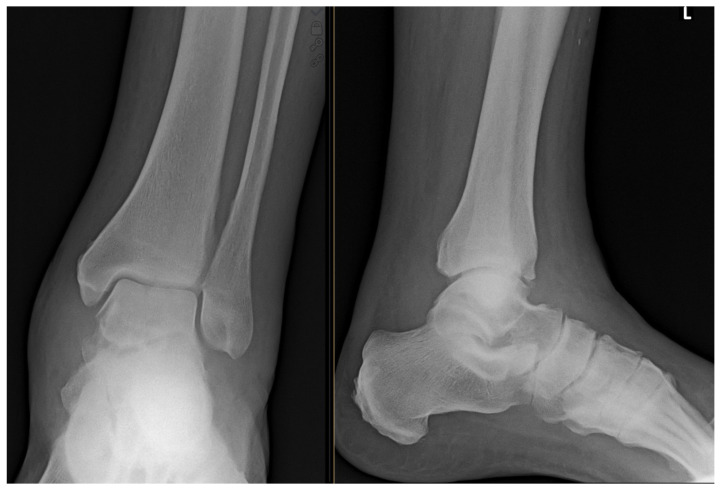
Initial X-ray image of the left ankle. The image shows normal picture with no detectable osteolytic lesions.

The patient was a polymorbid 72-year-old man with type 2 diabetes with diabetic foot syndrome treated by oral antidiabetics, and cryptogenic organizing pneumonia treated by prednisone and methotrexate. He was examined on 6 February 2020, for a month-long sensation of warmth in the left leg and occasional fever. A puncture of the fluctuating mass in the affected area was performed and empirical therapy started with peroral (PO) clindamycin 300 mg every 6 hours. Since this therapy was ineffective, the patient was admitted on 10 February 2020, for incision and drainage of the abscess and initiation of an intravenous (IV) antibiotic (ATB) therapy. The initial CRP value was 53 mg/L; the white blood cell count was 13.77 × 10^9^/L. During the procedure, purulent fluid was drained and sent for a cultivation followed by a change in ATB therapy to amoxicillin/clavulanate 1.2 g every 8 h IV. In the postoperative period, the patient was afebrile, and the wound looked calm. No osteolytic lesions in the bone were observed on the initial X-ray (Figure 1).

After four days of cultivation, small grey colony-forming units (CFU) grew on the blood agar and modified chocolate agar. Gram and Ziehl–Neelsen stainings revealed the formation of irregular Gram-positive and acid-fast bacilli (AFB), respectively. The MALDI-TOF MS failed to accurately identify the causative agent. Subsequently, the amplification and sequencing of the 16S rRNA gene region was carried out, and susceptibility to appropriate ATBs was tested using the E-test and the microdilution method. The results were interpreted according to the Clinical and Laboratory Standards Institute [1]. The susceptibility to the following ATB was tested: amikacin, imipenem, clarithromycin, linezolid, doxycycline, tigecycline, ciprofloxacin, moxifloxacin, and trimethoprim-sulfamethoxazole. ATB therapy was changed according to preliminary results to doxycycline 100 mg PO every 12 h.

The sequence evaluation of the part of 16S rRNA gene revealed *M. abscessus/chelonae* DNA with a lower identification score of 93%. (Supplementary Figure S1). To accurately determine the isolate, amplification of the *rpo*B gene was followed by sequencing and analysis using the BLAST (Basic Local Alignment Search Tool) database. From this analysis, *M. chelonae* DNA was clearly identified with a probability of 99.9% (Supplementary Figure S2).

The susceptibility of the strain to tigecycline (0.125 mg/L), tetracycline (0.5 mg/L), amikacin (16 mg/L), clarithromycin (2 mg/L), and moxifloxacin (1 mg/L) was proven, with resistance to imipenem (>32 mg/L), linezolid (32 mg/L), ciprofloxacin (4 mg/L), and trimethoprim-sulfamethoxazole (256 mg/L).

**Figure 2:**
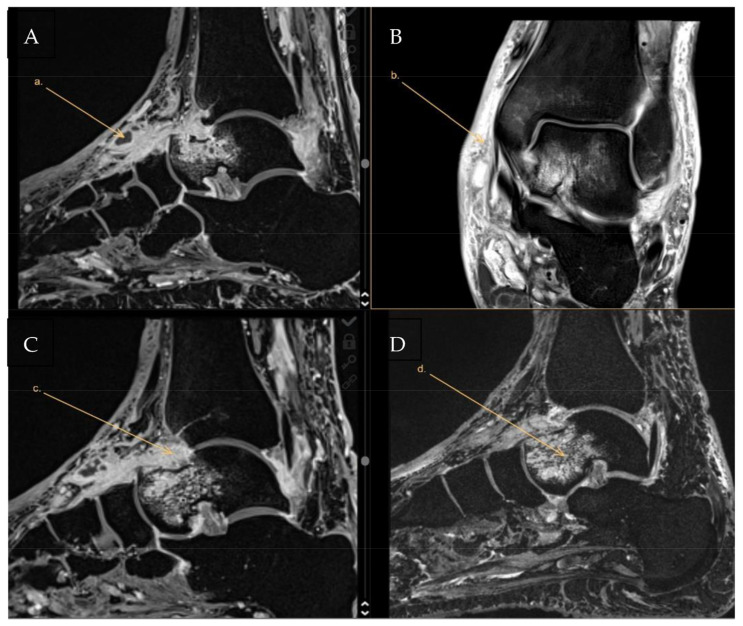
MRI of the affected left ankle on day 8. Arrows in the individual panels show (**A**) multiple small abscesses in the medial side of the feet, (**B**) edema of the tendons, (**C**) arthritis of the TC joint, and (**D**) osteomyelitis of the talus.

The subsequently performed MRI (day 8 of hospitalization) of the affected left ankle showed multiple small abscesses in the medial side of the feet, edema of the tendons, arthritis of the talocalcaneal (TC) joint, and clear signs of the osteomyelitis of the talus (Figure 2). According to Waldvogel et al. [2], this case can be thus classified as vascular insufficiency-associated osteomyelitis (patient with diabetes mellitus).

After that, the antibiotic therapy was changed to the IV combination of tigecycline 50 mg per 12 h, amikacin 1 g once a day, and moxifloxacin 400 mg once a day. A week later, a surgical revision of the soft tissues in the medial part of the tarsus was performed. Intraoperatively, a bone defect in the neck of the talus was found, followed by an excochleation of the osteomyelitic cavity and necrotic mass removal. During the revision, samples of the granulation tissue from the original scar, TC joint lining tissue, and talus bone tissue were collected for microbiological and histological examination. The resulting cavity was filled with gentamicin–collagen sponge (Garamycin SHWAMM, Eusa Pharma Ltd., Oxford, UK).

**Figure 3:**
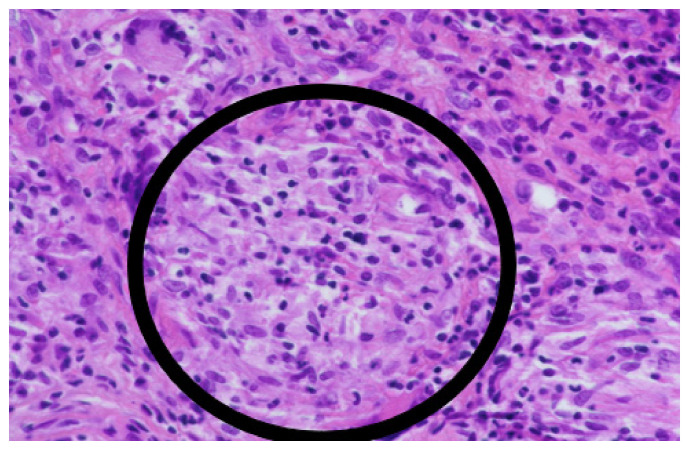
Epitheloid histiocytes and multinucleated giant cells of Langerhans’ type forming specific granulomatous tissue. Some neutrophils in the center of granulomas can be identified. However, no caseous necrosis is present (400×).

**Figure 4:**
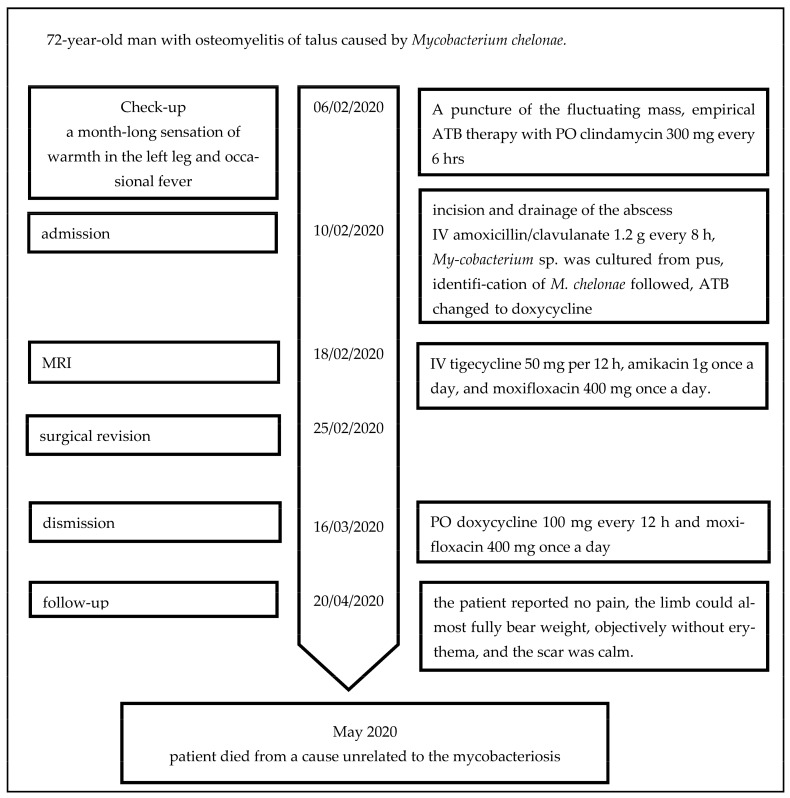
The timeline of the case.

The culture yielded a negative result, but the histological examination showed granulomatous inflammation (Figure 3) and the presence of AFB in the talus and adjacent tissue. A conservative approach was selected due to the patient’s comorbidities. The patient was treated for a total of four weeks with the IV combination of tigecycline, amikacin, and moxifloxacin. During this therapy, the status of the affected limb improved, and the patient was discharged home in good condition and with a PO combination of doxycycline 100 mg every 12 h and moxifloxacin 400 mg once a day. The duration of ATB therapy was tentatively determined to last at least for 6–12 months. The timeline of the therapy is depicted in Figure 4.

During the check-up at 2 months after the beginning of the therapy, the patient reported no pain, and the limb could almost fully bear weight. Objectively, there was no erythema, and the scar was calm. The next scheduled check-up was not performed due to the fact that the patient died from a cause unrelated to the mycobacteriosis that he had been treated for.

## 2. Literature Review and Discussion

*Mycobacterium (M.) chelonae* is classified as a rapidly growing mycobacterium (RGM) commonly found in soil, water, biofilm, and aquarium fish. Primarily, it is considered nonpathogenic, but under certain conditions, e.g., in immunosuppressed patients, in the setting of a damaged skin or mucous membrane, it can cause an infection [3,4]. *M. chelonae* causes mainly disseminated diseases in immunocompromised patients and soft tissue infections, but phlegmons, surgical wound infections, and keratitis are also reported. However, it has been rarely proven as an etiologic agent in osteomyelitis [5,6,7,8].

Osteomyelitis caused by RGM is a rare, very serious, and difficult-to-treat disease [9,10]. It usually occurs in immunocompromised patients [5,11] or in patients with wounded skin caused by an injection, tattooing or surgery, and a subsequent mycobacterial inoculation [8,12,13,14,15,16]. In the case described here, the infection was probably caused by *M. chelonae* entering through a small wound on the leg, likely during gardening, about half a year before the appearance of clinical manifestations. Exposure to soil is one of the risk factors for extrapulmonary mycobacterioses [17,18]. 

Gardening (in addition to farming, fishing, and swimming) has been previously described as an activity during which commonly found nontuberculous mycobacteria (NTM), including RGM, can infect or colonize a wound caused by the activity itself [12,19]. The exposure of the damaged skin to the soil is the main predisposing factor reported in 42% of patients with osteomyelitis caused by NTM, in whom previous immunosuppression was reported in 34% and surgery in 27% [12]. Usually, such a small local involvement manifests itself after longer periods with an erythema, abscess, and, subsequently, formation of fistulas. These skin infections can then spread locally and affect surrounding tissues as well, causing the inflammation of muscles, tendons, joints, and bones [6,12,14]. In immunocompromised patients, a hematogenous dissemination is common, often complicated by the infection of the vertebral body [20], but the osteomyelitis of a toe has also been described [21].

The most common etiological agents of musculoskeletal mycobacterioses are *M. avium-intracellulare* (MAC) complex members, RGM, and *M. marinum* [14,22,23]. Specifically, of the RGM, *M. abscessus*, *M. chelonae,* and *M. fortuitum* are found [24,25,26]. Hands and wrists, knees, the spine, legs, elbows, shoulders, and ankles represent the main affected areas. MAC members are usually the cause of osteomyelitis in generalized mycobacterioses of immunocompromised patients [27].

The laboratory diagnosis of NTM presents a challenge. For the isolation of mycobacteria, different procedures, including a decontamination prior to the culture, special culture media, and a prolonged culture period, are required compared to the conventional culture of more frequent and common bacteria [28]. Therefore, the clinical laboratories should be aware of the possibility of mycobacterial infections [3]. The choice of the sample and the administration of antibiotics before collection can negatively affect the result of culture. In particular, swabs have a low sensitivity and a high risk for contamination. Therefore, pus or tissue samples should be preferred [29]. In the case described here, pus was cultured before the administration of antibiotics. However, the mycobacterial etiology was also not considered at the beginning. The finding of *M. chelonae* was successful due to the prolonged conventional microbiological culture. According to the standard procedure in our laboratory, the culture of samples from chronic defects, fistulas, and other selected materials was extended up to 7 days, during which the growth of RGM was presented not only on special media, but also on blood agar [30].

Identification of mycobacteria using MALDI-TOF MS or molecular methods using commercially available kits for the most frequently occurring mycobacteria can be used (i.e., GenoType^®^ Mycobacterium CM/AS; Hain Lifescience GmbH, Nehren, Germany). Methods based on DNA sequencing are usually available only in larger laboratory facilities. In our case, the identification using MALDI-TOF MS did not yield a reliable result, and thus, DNA sequencing by capillary electrophoresis of conservative DNA regions was performed. First, the amplification and sequencing of the 16S rRNA region permitted the identification of the strain only to the level of the *M*. *abscessus/chelonae* complex. Only the subsequent amplification and sequencing of a part of the *rpo*B gene yielded a clear determination of the species *M. chelonae*, with a percent identity of 99%. These results are similar to other studies, which have shown that *rpo*B gene sequencing is a robust and more reliable tool compared to the 16S rRNA region sequencing, for the accurate identification of not only the known mycobacterial species, but also new ones [31,32].

The therapy of mycobacterioses is challenging due to the frequent antibiotic and multidrug resistance of these pathogens. There are no clinical controlled trials available to compare individual therapeutic strategies. The treatment is usually either based on the experience gained from case reports, or on the results of the ATB susceptibility profile of the particular mycobacterial strain. In addition, results acquired mainly during the therapy of pulmonary mycobacterioses have been used [3]. The accurate identification of the species of the causative mycobacterial agent is thus essential for the selection of the appropriate ATB therapy. It is recommended to start the treatment with a combination of an aminoglycoside with two other effective ATBs. It is necessary to choose carefully from the already-narrowed armamentarium in order to minimize the adverse effects of individual drugs or their combinations [33]. For mycobacterioses affecting the musculoskeletal system, at least 6 months of combination drug therapy (to prevent the development of antibiotic resistance) is advocated, with an extension of up to 12 months in more complicated and severe infections. However, the optimal duration of antibiotic treatment remains unclear [3,33].

The basic component of the combination therapy is clarithromycin. However, it is known that *M. abscessus* can develop a resistance to macrolides based on the presence of the *erm*(41) gene for the inducible resistance, and therefore, the susceptibility to macrolides should always be tested with an incubation period extended to 14 days [34]. At the time when osteomyelitis was diagnosed in our patient, the mycobacterial isolate had not yet been reliably determined, since the sequencing of 16S rRNA showed the species *M*. *abscessus* and *M. chelonae* with equal probability. Based on this result, the combination therapy including amikacin, tigecycline, and moxifloxacin, but without clarithromycin, was initiated. 

Unlike *M. abscessus, M. chelonae* is usually susceptible to macrolides. The isolate described in this report remained sensitive to clarithromycin in vitro after a 14-day incubation period. However, because the previously initiated ATB combination therapy had worked very well with proven positive clinical effects, the ATB combination was not changed. Even after the transition to oral therapy, the ATB combination continued without macrolide. The combination of doxycycline and moxifloxacin proved effective and continued after the follow-up. The duration of therapy was tentatively set at 6–12 months, with the possibility of an extension, which is usually required in immunocompromised patients and in patients without radical surgery [35]. Unfortunately, the patient died due to an unrelated cause before the infection could be completely cured.

## 3. Materials and Methods

A purulent fluid from the abscess was cultivated on blood agar, modified chocolate agar, MacConkey agar, and Schaedler agar (Thermo Fisher Scientific, Basingstoke, UK) at 37 °C under aerobic and anaerobic condition. MALDI-TOF MS was performed using MALDI Biotyper (Bruker-Daltonics Billerica, MA, USA).

Identification of isolated strain was performed by molecular genetic methods. Nucleic acids were isolated directly from the suspension of bacterial isolate using the QIAamp DNA Mini Kit (Qiagen, Hilden, Germany). At first, identification was performed by 16S rRNA region amplification and sequencing with universal primers complementary to four conserved regions alongside two hypervariable sequences, V3 (5′-CCAGACTCCTACGGGAGGCAG-3′) and V6 (5′-ACATTTCACAACACGAGCTGACGA-3′), as described before [36]. Amplification of single-copy gene encoding the RNA Polymerase β Subunit (*rpo*B) was performed using primers *rpo*B-F (5′- GGCAAGGTCACCCCGAAGGG-3′) and *rpo*B-R (5′-AGCGGCTGCTGGGTGATCATC-3′) [37]. The sequences were then obtained using ABI 3500 (Genetic Analyzer, Applied Biosystems/Thermo Fisher Scientific, Foster City, CA, USA) and subsequently analyzed with Bionumerics 7.6.2 (Applied Maths, Ghent, East Flanders, Belgium). 

Antibiotic (ATB) susceptibility examination was performed by the broth microdilution method. MIC values of amikacin, imipenem, clarithromycin, linezolid, doxycycline, tigecycline, ciprofloxacin, moxifloxacin, and trimethoprim-sulfamethoxazole were performed using the broth microdilution method in 96-well microtiter plates. The cation-adjusted Mueller-Hinton (Becton Dickinson, Sparks, MD, USA) was used and the results were interpreted according to the Clinical and Laboratory Standards Institute [1].

## 4. Conclusions

*M. chelonae* belongs among rare causative agents of osteomyelitis. Laboratory diagnostics is difficult and for the bacterial culture to be successful, it requires extending the incubation period for up to 7 days. For the exact determination, molecular typing needs to be subsequently performed. The comprehensive approach, including the surgical intervention and administration of effective ATB, is essential for the infection management. The ATB therapy is recommended to last 6–12 months and the selection of effective ATB is enabled only by the correct identification of the mycobacterial species.

## Data Availability

Data are available on request due to the ethical restrictions.

## Supplementary Materials

The following supporting information can be downloaded at: https://www.mdpi.com/article/10.3390/antibiotics12010097/s1, Figure S1: Sequence of 16S rRNA region of the mycobacterial isolate; Figure S2: Sequence of single copy gene encoding the *rpo*B gene of the mycobacterial isolate.
