# Supplementary material for: A Rare Case of Osteomyelitis of an Ankle Caused by Mycobacterium chelonae
Source: Antibiotics (Basel). 2023 Jan 6;12(1):97. doi: 10.3390/antibiotics12010097 (PMC9854486; doi:10.3390/antibiotics12010097)
Supplement: Supplementary file 1 [file antibiotics-12-00097-s001.zip › antibiotics-2118052-supplementary.pdf]

Supplementary Figures.

CGGGAGGCAGCAGTGGGGAATATTGCACAATGGGCGCAAGCCTGATGCAGCGACGC
CGCGTGGAGGGATGACGGCCTTCGGGTTGTAAACCTCTTTCAGTAGGGACGAAGCGA
AAGTGACGGTACCTACAGAAGAAGGACCGGCCAACTACGTGCCAGCAGCCGCGGTA
ATACGTAGGGTCCGAGCGTTGTCCGGAATTACTGGGCGTAAAGAGCTCGTAGGTGGT
TTGTCGCGTTGTTCGTGAAAACTCACAGCTTAACTGTGGGCGTGCGGGCGATACGGG
CAGACTAGAGTACTGCAGGGGAGACTGGAATTCCTGGTGTAGCGGTGGAATGCGCA
GATATCAGGAGGAACACCGGTGGCGAAGGCGGGTCTCTGGGCAGTAACTGACGCTG
AGGAGCGAAAGCGTGGGTAGCGAACAGGATTAGATACCCTGGTAGTCCACGCCGTA
AACGGTGGGTACTAGGTGTGGGTTTCCTTCCTTGGGATCCGTGCCGTAGCTAACGCAT
TAAGTACCCCGCCTGGGGAGTACGGTCGCAAGACTAAAACTCAAAGGAATTGACGG
GGGCCCGCACAAGCGGCGGAGCATGTGGATTAATTCGATGCAACGCGAAGAACCTT
ACCTGGGTTTGACATGCACAGGACGTACCTAGAGATAGGTATTCCCTTGTGGCCTGT
GTGCAGGTGGTGCATGGCTGTCGTCAGCT

Supplementary Figure S1. Sequence of 16S rRNA region of the mycobacterial isolate. Analysis in BLAST and SepsiTest™ BLAST showed indeterminate result for *M. chelonae* and *M. abscessus*.

GAAGGGTGAAACCGAGCTGACCCCCGAGGAGCGCCTGCTGCGTGCCATCTTCGGTG
AGAAGGCCCCGCGAGGTTCGCGACACCTCCCTCAAGGTGCCGCACGGTGAGTCCGG
CAAGGTCATCGGCATCCGCGTCTTCTCGCGTGATGACGACGACGACCTGCCCGCCGG
CGTGAACGAGCTCGTTCGCGTGTACGTCGCGCAGAAGCGCAAGATCTCCGACGGTG
ACAAGCTGGCCGGACGCCACGGCAACAAGGGCGTCATCGGCAAGATCCTGCCCGTC
GAGGACATGCCGTTCCTGCCCGATGGCACCCCGGTGGACATCATCCTGAACACCCAC
GGTGTGCCGCGTCGTATGAACATCGGCCAGATCCTGGAGACCCACCTGGGGTGGGTG
GCCAAGACCGGCTGGAACATCGAGGGCAACCCCGAGTGGGCGCAGAACCTCCCCG
AGGATCTGCAGTCGGCCCCGGCCGACACCCGCACGGCCACCCCGGTGTTCGACGGC
GCCCGCGAGGAGGAGCTGACCGGACTGCTGTCCTCGACGCTGCCCAACCGGGACGG
CGAGGTCATGGTGGACGGTGACGGCAAGGCACGGCTGTTCGACGGCCGTAGCGGTG
AGCCGTTCCCGTACCCGGTGACCGTCGGCTACATGTACATCCTGAAGCTGCACCACT
TGGTCGACGACAAGATTCACGCGCGTTCGACCGGTCCGTACT

Supplementary Figure S2. Sequence of single copy gene encoding the *rpo*B gene of the mycobacterial isolate. The BLAST analysis identified rpoB gene of *M. chelonae* with identification score of 99.86%.
